# Acceleration of reaction in charged microdroplets

**DOI:** 10.1017/S0033583515000086

**Published:** 2015-11

**Authors:** Jae Kyoo Lee, Shibdas Banerjee, Hong Gil Nam, Richard N. Zare

**Affiliations:** 1 Department of Chemistry, Stanford University, Stanford, CA 94305, USA; 2 Center for Plant Aging Research, Institute for Basic Science (IBS), DGIST, Daegu 711-873, Republic of Korea; 3 Department of New Biology, DGIST, Daegu 711-873, Republic of Korea

**Keywords:** Electospray ionization, droplet fusion, isoquinoline, cytochrome c, maltose, kinetics

## Abstract

Using high-resolution mass spectrometry, we have studied the synthesis of isoquinoline in a charged electrospray droplet and the complexation between cytochrome *c* and maltose in a fused droplet to investigate the feasibility of droplets to drive reactions (both covalent and noncovalent interactions) at a faster rate than that observed in conventional bulk solution. In both the cases we found marked acceleration of reaction, by a factor of a million or more in the former and a factor of a thousand or more in the latter. We believe that carrying out reactions in microdroplets (about 1–15 μm in diameter corresponding to 0·5 pl – 2 nl) is a general method for increasing reaction rates. The mechanism is not presently established but droplet evaporation and droplet confinement of reagents appear to be two important factors among others. In the case of fused water droplets, evaporation has been shown to be almost negligible during the flight time from where droplet fusion occurs and the droplets enter the heated capillary inlet of the mass spectrometer. This suggests that (1) evaporation is not responsible for the acceleration process in aqueous droplet fusion and (2) the droplet–air interface may play a significant role in accelerating the reaction. We argue that this ‘microdroplet chemistry’ could be a remarkable alternative to accelerate slow and difficult reactions, and in conjunction with mass spectrometry, it may provide a new arena to study chemical and biochemical reactions in a confined environment.

## Introduction

Most chemical reactions are conventionally run in bulk solvent. Our group has been developing a so-called ‘microdroplet fusion mass spectrometry’ that would allow observation of early events in chemical and biochemical processes (Lee *et al*. 2015). During the course of our study, we employed the redox reaction between 2, 6-dichlorophenolindophenol (DCIP) and ascorbic acid and found that the reaction rate was over a 1000-fold higher than the previously reported rate in bulk solution. Recently, a few other groups have explored reactions in droplets using electrospray ionization (ESI) (Badu-Tawiah *et al*. 2012; Bain *et al*. 2015; Banerjee, 2013; Banerjee *et al*. 2011, 2013; Fallah-Araghi *et al*. 2014; Girod *et al*. 2011; Müller *et al*. 2012; Perry *et al*. 2011, 2012). These groups have also reported the acceleration of chemical reaction rates in microdroplets, in both charged droplets surround by air and non-charged aqueous droplets in oil. Questions arise: How general is the acceleration of chemical reactions in micro-droplets? To what degree can the reaction be accelerated? What types of reactions are applicable for accelerating the reaction in microdroplets? What are the mechanisms of the different reaction rates in the bulk- and microdroplet-chemistry?

**Fig. 1:**
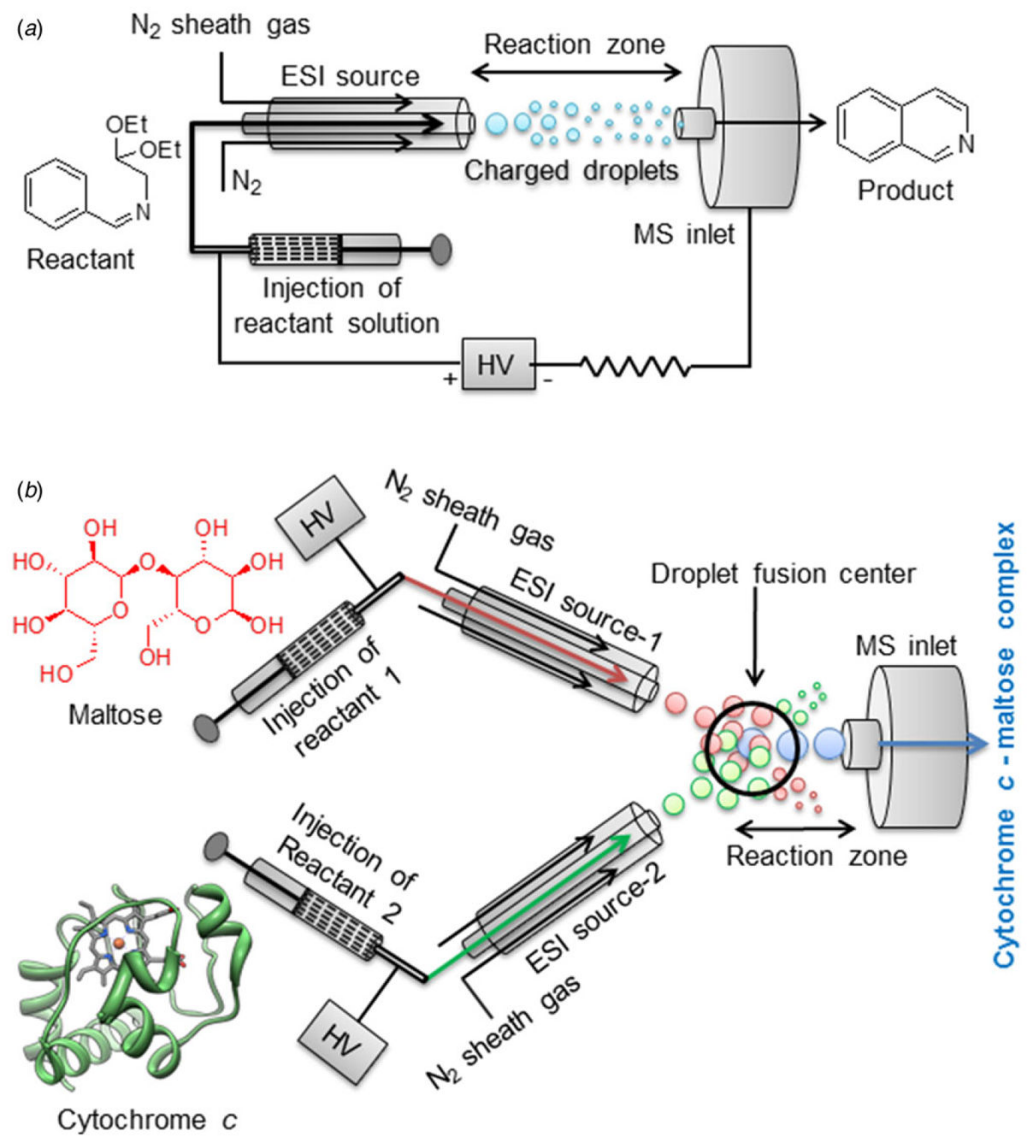
Schematic diagrams of the experimental setup used in our study of ‘microdroplet chemistry’. The upper panel (*a*) shows the electrospray assisted synthesis of isoquinoline, and the lower panel (*b*) shows the droplet fusion mass spectrometry to study the complexation between maltose and cytochrome *c*.

To address some of these questions, we have investigated two different reactions in microdroplets. One is a chemical reaction with covalent bond rearrangement and the other is noncova-lent protein–ligand interaction. First, the well-known chemical reaction of Pomeranz–Fritsch synthesis of isoquinoline (Bobbitt & Bourque, 1987; Gensler, 2004; Li, 2006) was examined in microdroplets generated by the electrospray process (Fig. 1*a*). We have also explored the kinetics of cytochrome *c* and maltose interaction in microdroplets with the droplet fusion mass spectrometry we developed (Fig. 1*b*). We report more than a million fold increase of the reaction rate of the Pomeranz–Fritsch synthesis of isoquinoline, and more than a 1000-fold increase of the rate of cytochrome *c*–maltose binding compared with that in bulk solution. We also consider the evaporation of microdroplets and present arguments that evaporation is not the principal cause for acceleration of reaction rates in the case of fused aqueous droplets.

## Materials and methods

### Chemicals and sample preparation

Horse heart cytochrome *c*, maltose, benzaldehyde, aminoacetaldehyde diethyl acetal, and *m*-nitrobenzyl alcohol (*m*-NBA) were purchased from Sigma (St. Louis, MO). HPLC grade methanol, *N,N*-dimethylformamide (DMF), acetonitrile (ACN), and water were purchased from Fisher Scientific (Nepean, ON, Canada).

### Electrospray-assisted Pomeranz–Fritsch synthesis of isoquinoline

**Scheme 1:**
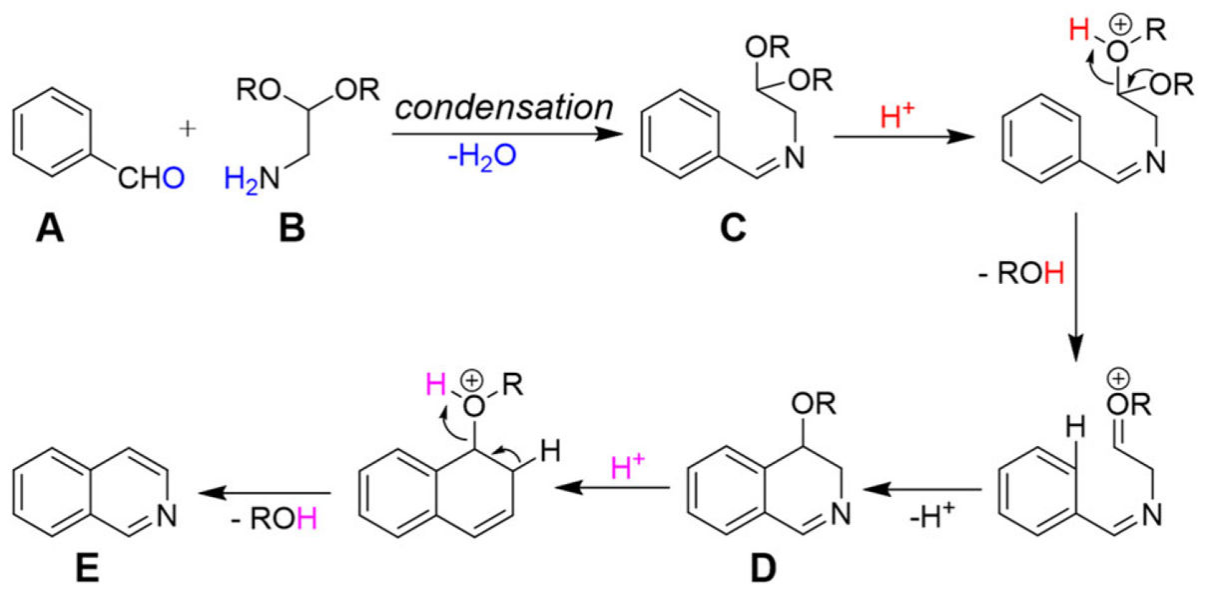
The plausible mechanism of Pomeranz–Fritsch reaction.

1 mmol (110 μl) benzaldehyde was mixed with 1 mmol (145 μl) aminoacetaldehyde diethyl acetal and heated at 100 °C for 2 h to form (*Z*)-*N*-benzylidene-2,2-diethoxyethanamine precursor (**C**, in Scheme 1). Then 5 μl aliquot of the above precursor (**C**) was dissolved in different solvents (methanol, water, 1:1 DMF–ACN mixture, and 1% *m*-NBA in water) and electrosprayed in positive ion mode (+5 kV) at a flow rate of 15 μl min^−1^ through silica tubing (100 μm i.d.) with a coaxial sheath gas flow (N_2_ at 120 psi). The mass spectrometer inlet capillary temperature was maintained at approximately 275 °C, and capillary voltage was kept at 44 V. The spray distance (the on-axis distance from spray tip to the entrance of the heated capillary; see Fig. 1*a*) was kept at 1·5 cm. All experiments were carried out under identical conditions to detect the product by a high-resolution mass spectrometer (Thermo Scientific LTQ Orbitrap XL Hybrid Ion Trap-Orbitrap mass spectrometer).

### Microdroplet fusion mass spectrometry of cytochrome c–maltose complexation

Thermo Scientific LTQ Orbitrap XL Hybrid Ion Trap-Orbitrap mass spectrometer was used for the cytochrome *c*–maltose binding studies in fused droplets. Two ESI-like spray sources are equipped with an X–Y–Z micro positioning linear and angular stage for accurate alignment of the two streams of droplets (See Fig. 1*b*). This alignment is important for ensuring fusion of most of the incident droplets and to maintain a linear trajectory toward the mass spectrometer inlet. The best alignment was acquired with the angle between two crossed droplet streams at 78°, which showed the highest probability of droplet fusion and straight trajectories of the fused droplets to the inlet of the mass spectrometer. Two aqueous solutions of analytes (cytochrome *c* at a concentration of 100 μM and maltose at a concentration of 100 mM) were injected from the two ESI sources with a syringe pump (Harvard Apparatus, Holliston, MA) at a flow rate of 30 μl min^−1^ in positive ion mode. The heated capillary temperature was maintained at approximately 275 °C, and the ion-spray voltage was kept at +5 kV. For measurement of the size and velocities of the fused droplets over a distance *x*, we used a high-speed optical camera (Phantom v1610, Vision Research, Wayne, NJ).

## Results and discussion

### Pomeranz–Fritsch synthesis of isoquinoline in charged microdroplets

**Fig. 2:**
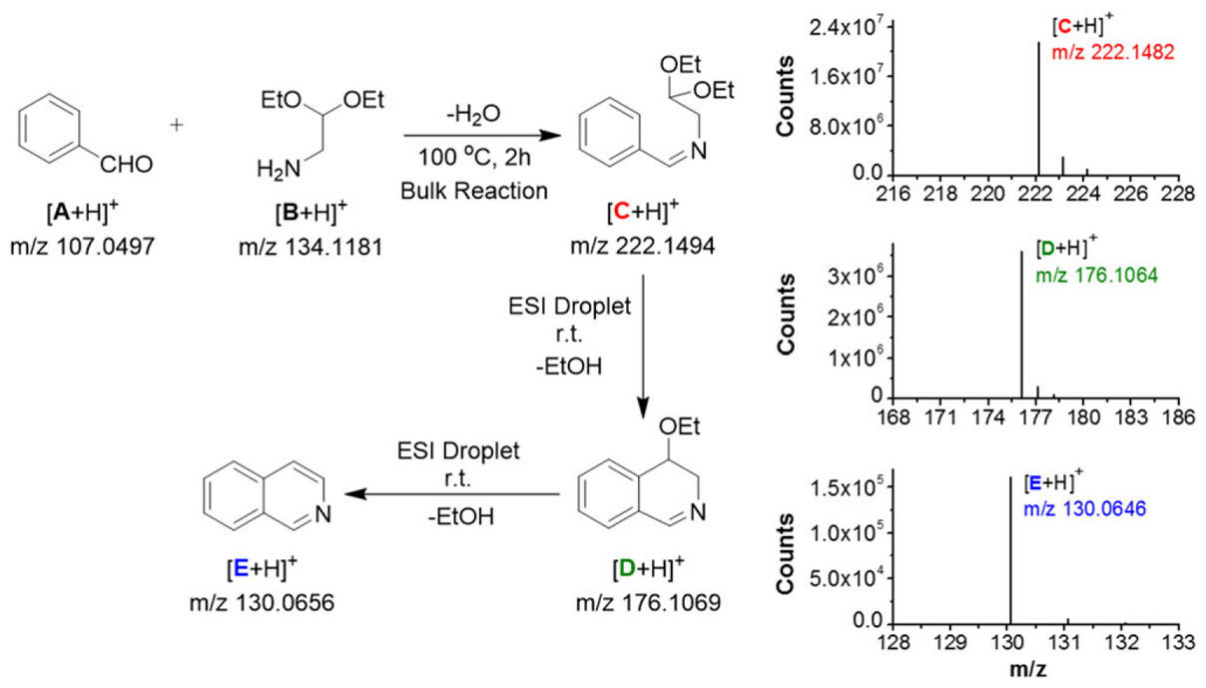
Pomeranz–Fritsch synthesis of isoquinoline in the charged droplet produced by electrospray process. The left panel shows the two step synthesis of isoquinoline that we followed in the present study. In the first step, the conventional bulk reaction method was used to synthesize the precursor imine **C**. Then in the second step, the precursor **C** was injected from methanolic solution through an on-axis electrospray source, in positive ion mode, to form charged droplets encapsulating the precursor **C**, which was then converted into isoquinoline (**E**) inside the charged droplet *via* intermediate **D**. Each protonated species (precursor **C**, intermediate **D**, and product **E**) were detected and characterized by a high resolution orbitrap mass spectrometer (see the spectra in the right panel; solvent: methanol). The theoretical values of *m*/*z* (see left panel) are in good agreement with that experimentally observed (see the right panel).

The charged microdroplet (1–2 μm in diameter) produced by ESI process in positive ion mode is highly acidic and the pH of the droplet continuously decreases during the evolution period (repeated solvent evaporation and Coulomb fission in conventional ESI) of the droplet (Banerjee & Mazumdar, 2012; Fenn, 1993; Kebarle & Tang, 1993). Here we attempt to induce a typical acid-catalyzed reaction e.g. Pomeranz–Fritsch synthesis (Bobbitt & Bourque, 1987; Gensler, 2004; Li, 2006) of isoquinoline in a charged droplet to investigate whether high proton density in the electrospray droplet surface can accelerate the reaction in a confined space of a continuously evaporating droplet. In bulk solvent, this synthesis is believed to proceed in two steps (see Scheme 1), the condensation of aldehyde (**A**) and amine (**B**) to form the imine (**C**), followed by acid-induced ring closure *via* intermediate (**D**) to yield isoquinoline (**E**) (Li, 2006, 2009). The second step requires a high acid concentration (typically ~70% sulfuric acid) as well as a long reaction time, ranging from hours to days (Gensler, 2004). We prepared separately the imine (**C**), which was dissolved in methanol, and electrosprayed into a high-resolution mass spectrometer as depicted in Fig. 1*a*. In sharp contrast to the behavior in bulk solution, we found the production of isoquinoline from (**C**) in charged microdroplets (see Fig. 2), even though the average lifetime of the charged droplet is of the order of milliseconds and no acid has been added to induce the reaction. Moreover, we detected the intermediate (**D**), which was proposed earlier but no direct experimental evidence of its existence was presented. The acceleration of the reaction rate is estimated to be roughly more than a factor of a million, based on the yield and ionization efficiency of (**E**). This remarkable behavior of reactions in electrospray droplets appears to us to be highly promising for preparative scale synthesis of important isoquinoline-based organic compounds (e.g. fine chemicals) on a short timescale. Isoquinoline is a precursor material of many biologically active compounds such as anesthetics, antihypertension agent, antifungal agent, disinfectants, and many other drugs (Waldvogel, 2005).

We also have investigated the effects of droplet solvent composition (see Table 1) by monitoring the progress of the reaction of (**C**) in different solvents (of microdroplets). We measured the absolute intensities (counts) of the individual species (reactant, intermediate, and product). Our experimental data (Table 1) suggest that the reaction efficiency in microdroplets depends on cumulative effects of multiple properties of the droplet such as evaporation, charge accumulation, average lifetime, polarity of the droplet, etc. The maximum reaction progress was observed in the droplet produced from 1% *m*-NBA in water. The *m*-NBA is popularly known as a supercharging agent in the electro-spray process (Iavarone & Williams, 2003; Lomeli *et al*. 2010; Sterling *et al*. 2010). It also enhances the average droplet lifetime because of its very low volatility (vapor pressure given in Table 2). Thus, the confinement of the reagent (**C**) in a highly charged, comparatively long-lived droplet might help the reaction to occur to a greater extent. On the contrary, when ACN–DMF mixture was used as the solvent, we observed the lowest reaction efficiency (see Table 1), although low volatile DMF (vapor pressure data given in Table 2) (Lide, 1996) enhances the droplet lifetime. The possible reason of this low reaction efficiency in the presence of DMF might be caused by low surface-charge (protons) accumulation, which is largely guided by Rayleigh limit charging (dependence of surface charge density on the surface tension of the droplet; the surface tension data have been listed in Table 2) as given by Eq. (1) (Banerjee & Mazumdar, 2012; Kebarle & Tang, 1993; Rayleigh, 1882).

(1) $$Z_{R}\;e=8\pi \;{\left(\varepsilon_{0}\;\gamma \;R^{3}\right)}^{1/2},$$

where *Z_R_* is the charge limit, *e* is the elementary charge, *R* is the radius of the charged droplet, *γ* is the surface tension, and *ε*_0_ is the permittivity of the surrounding medium.

Likewise, the efficiency of product formation in methanol and water droplet is roughly similar under the present experimental conditions (see Table 1) possibly by the combined effects of vapor pressure and surface tension (see Table 2) as discussed above. However, a detailed study of possible effects of droplet solvent composition on the reaction rate enhancement needs to be undertaken at different instrumental conditions to extract more comprehensive information on the mechanism of reaction acceleration in the droplet compared with that in conventional bulk phase. Nevertheless, this preliminary observation of superfast reaction in the microdroplet is quite fascinating. It might open a new dimension in the field of synthetic organic chemistry, where water would be used as an environmentally benign solvent (green chemistry).

### Reaction kinetics of cytochrome c and maltose binding in microdroplets

**Fig. 3:**
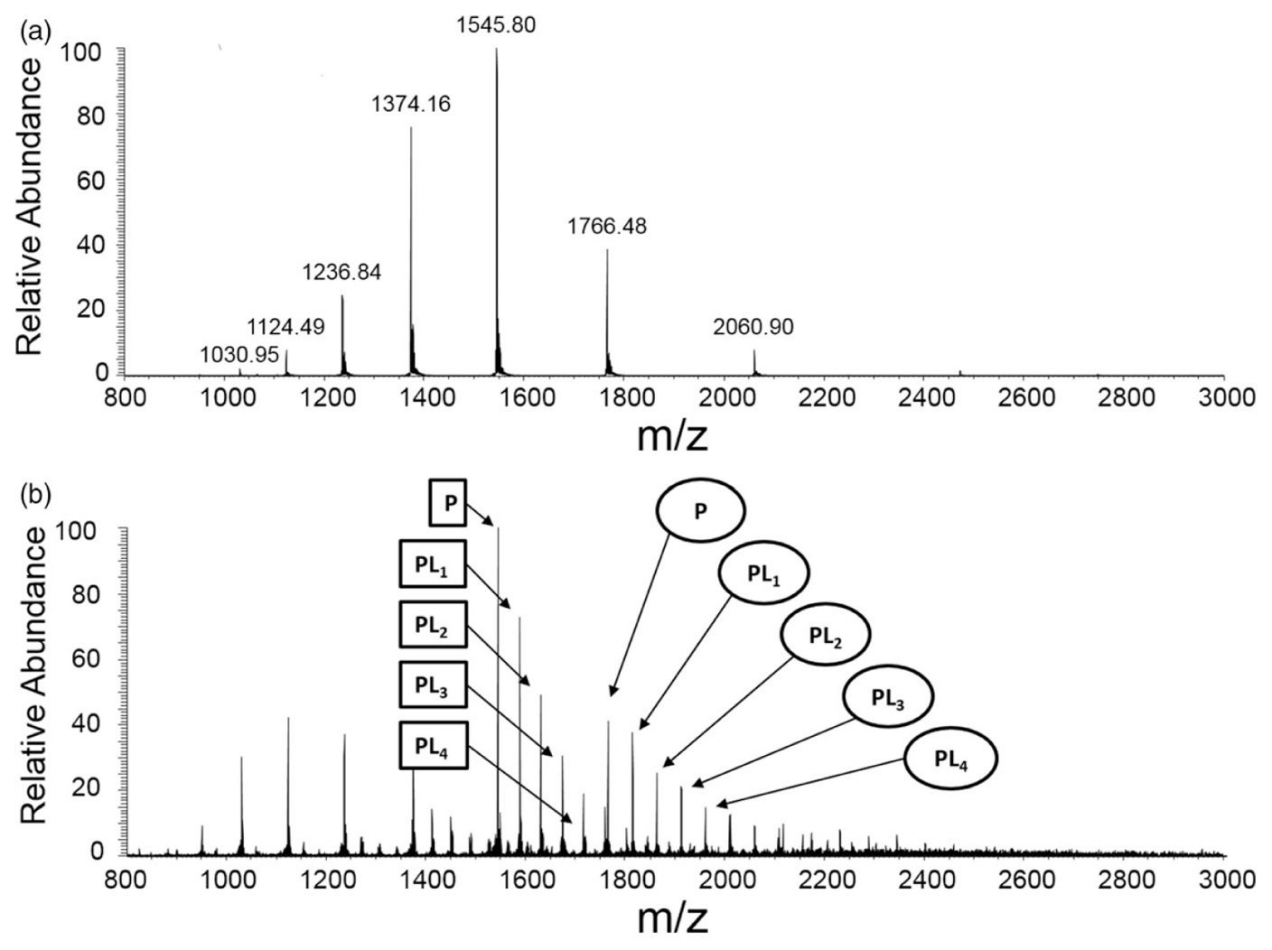
ESI-mass spectra of (*a*) cytochrome *c* (100 μM) and (*b*) cytochrome *c* (100 μM) incubated with maltose (100 mM) for 20 min. The subscript *n* in PL*n* denotes the number of bound maltose to cytochrome *c* (square denotes +8 charge state and circle denotes +7 charge state).

**Fig. 4:**
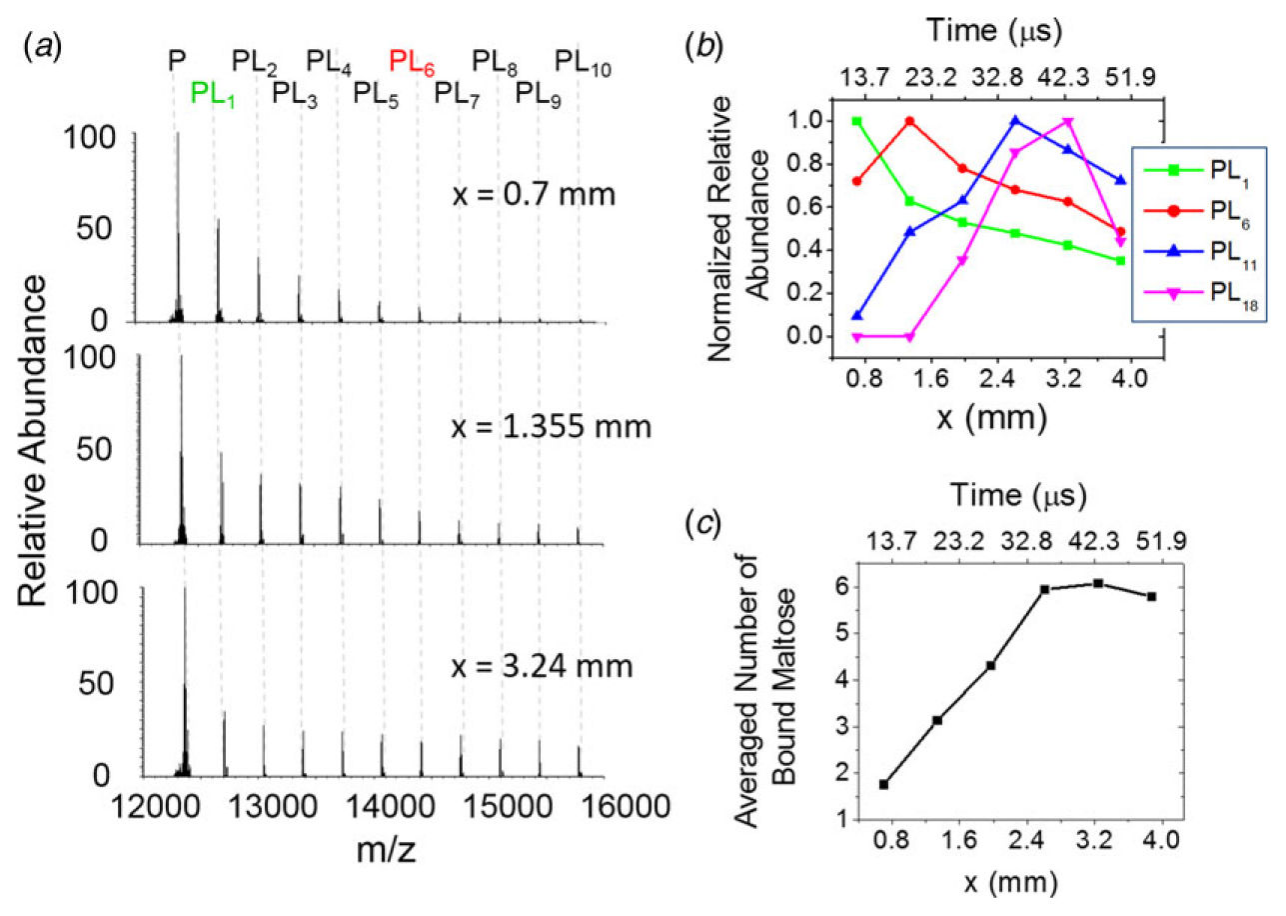
Kinetics of the binding of cytochrome *c* and maltose. (*a*) Deconvoluted mass spectra at different distances (*x*) with cytochrome *c* (100 μM) in one droplet source and maltose (100 mM) in the other source. The subscript *n* in PL*n* denotes the number of maltose bound to cytochrome *c*. (*b*) Normalized relative abundances of cytochrome *c* with different number of bound maltose (green square: PL1, red circle: PL_6_, blue triangle up: PL_11_, magenta triangle down: PL_18_). The normalized factor for each plot for PL_1_, PL_6_, PL_11_, and PL_18_ is ×1, ×4·7, ×11·7, and ×17·4, respectively. (*c*) Average number of bound maltose to cytochrome *c* as a function of distance *x* and reaction time. The axes on top of (*a*) and (*c*) show the converted reaction time from the corresponding distance.

Protein–sugar interaction are known to play important roles for cell–cell binding, cell–matrix interaction, migration of tumor cells, recognition of pathogens, and energy transport (Holgersson *et al*. 2005; Quiocho, 1986; Rauvala *et al*. 1981; Rudd *et al*. 2004). However, the kinetics of the sugar–protein interaction is not well understood. Here we investigated the kinetics of cytochrome *c* and maltose complexation since it was reported that the maltose possesses several hydroxyl groups noncovalently bound to cytochrome *c* though hydrogen bonding (Liu *et al*. 2012). In this report, we also show reaction rate acceleration on fusing together two aqueous electrospray droplets (see Fig. 1*b*), one containing cytochrome *c* and the other containing maltose, to form hydrogen-bonded noncovalent complexes in the fused droplet. Here we have used our previously developed droplet fusion apparatus (Lee *et al*. 2015) for investigating kinetics of this protein–ligand interaction. Figure 3 shows the conventional ESI-mass spectra of pure cytochrome *c* (upper panel) and cytochrome *c* premixed with maltose (lower panel). A distribution of multiply charged species comprising cytochrome *c* and different numbers of maltose *via* noncovalent hydrogen bonding interaction was detected when we electrosprayed the mixture of cytochrome *c* and maltose (see Fig. 3*b*). For kinetics analyses, two different droplets containing cytochrome *c* at 100 μM and maltose at 100 mM were fused while the distance *x* (from droplet fusion center to the heated capillary inlet of the mass spectrometer) was varied. A 1000-fold excess concentration of cytochrome *c* over maltose was used here to ensure binding of maltose to cytochrome *c* in the fused droplet that is travelling the distance *x* in very short timescale (tens of microseconds). Figure 4 shows the measured kinetics of cytochrome *c*–maltose binding. As the distance × increased from 0·7 to 3·875 mm in increments of 0·6 mm, the ion signal intensities corresponding to cytochrome *c* bound with higher numbers of maltose increased. The deconvoluted mass spectra at *x* = 3·875 mm (see Fig. 4*a*) indicated a maximum of 25 ligands (maltose) bound to cytochrome *c*. The signal intensity corresponding to cytochrome *c* with no maltose binding reached its maximum at *x* = 0·7 mm under the present experimental conditions, followed by a gradual decay over distance *x.* The ion signals corresponding to cytochrome *c* bound to 6, 11, and 18 maltose molecules reached their maxima at *x* = 1·335, 2·605, and 3·24 mm, respectively (see Fig. 4*b*), indicating the gradual occupation of maltose to available binding sites in cytochrome *c*. The average number of bound maltose molecules reached a plateau at 2·605 mm, corresponding to 35·2 μs in time (see Fig. 4*c*). The estimated association time constant for cytochrome *c* and maltose interaction was found to be 17·9 ± 8·6 *μ*s in the present study. The reported time constant for protein–sugar binding in bulk solvent is of the order of 10–100 ms (Miller *et al*. 1983). Therefore, this noncovalent reaction in the droplet has been accelerated by a factor of a thousand or more compared to that in bulk solution. It should be noted that this detailed information on the number and distribution of bound ligands to a protein demonstrates the power of mass spectrometric measurement of the protein–ligand interaction at a short timescale which is not readily available by population-averaged spectroscopic methods.

**Fig. 5:**
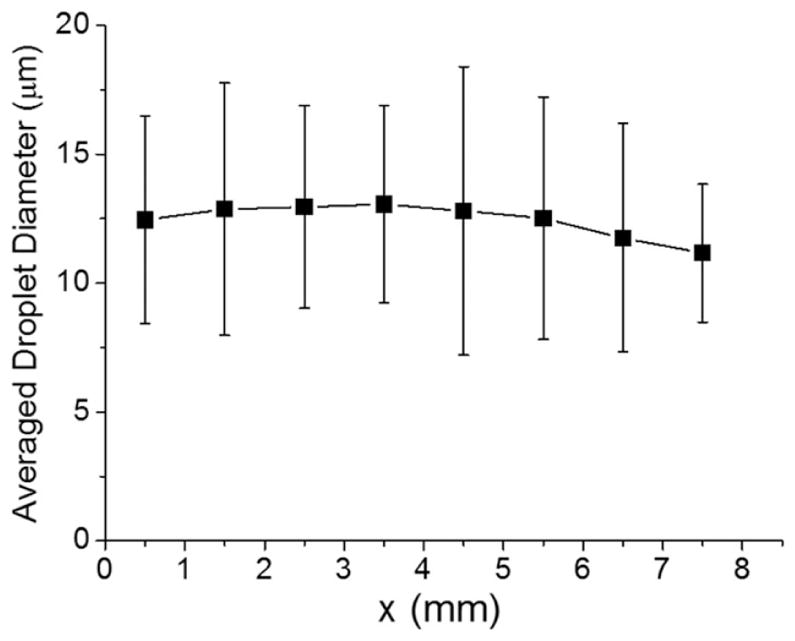
Average diameter of pure-water droplets in the microdroplet fusion mass spectrometry as a function of the distance (*x*). Few noticeable differences were observed in the average size of microdroplets up to the distance of about 7 mm from the droplet fusion center. All kinetic measurements shown in Fig. 4 are performed at distances of 4 mm or less.

To investigate the origin of acceleration of these noncovalent reactions in aqueous microdroplets, we have measured the sizes of fused droplets composed of pure water traveling from droplet fusion center to mass spectrometer inlet (see Fig. 1*b*) with a high-speed camera (see Fig. 5). We have not observed any significant decrease of the droplet size until the distance reaches to *x* = 7 mm. It needs to be emphasized that all kinetic measurements we make are performed for *x* − 4 mm. We do have large error bars on the measured droplet diameter, and the volume of the droplet varies as the cube of the diameter. Even if we imagine that the droplet diameter shrunk in size from 13 to 11 μm, this reduction corresponds to a volume change of less than 50%, which would cause the overall concentrations of reagents to increase by less than a factor of two. We believe that this concentration increase could contribute to but could not account for the marked enhancement in the reaction rate compared to bulk solution we have observed. Hence, we are led to conclude that confinement of reagents (cytochrome *c* and maltose) in a small volume might be the chief cause for reaction rate enhancement.

## Conclusion

We observe a remarkable acceleration in the reaction rate in microdroplets produced by electrospray and when two droplets containing different reagents are fused together. These findings are elevating our interest to conduct important reactions in liquid droplets (aerosols), which would provide a small confined volume for reagents to react in a faster rate than that in conventional bulk solution. The acceleration of the chemical reaction in microdroplets appears to be general regardless of reaction mechanisms, including specific covalent and nonspecifc noncovalent bonding. What causes the reaction acceleration in the microdroplet remains yet to be fully understood. The previous studies (Badu-Tawiah *et al*. 2012; Bain *et al*. 2015; Girod *et al*. 2011) emphasized that solvent evaporation of microdroplets plays an important role in accelerating the reaction. However, nearly constant droplet size in our droplet fusion mass spectrometric study suggests that the evaporation process may not be the only factor in accelerating the reaction. It is apparent that the reaction in a confined environment can occur in a different manner to that in a bulk environment. The chemistry at air–droplet surface interface (Griffith & Vaida, 2012; Jung & Marcus, 2007; Narayan *et al*. 2005, 2007) may play a special role in the reaction acceleration. However, a more detailed study is needed to investigate the possible mechanisms that bring about the acceleration of reaction in microdroplets.

‘Microdroplet chemistry’ is still in its infancy and raises our interest to explore this unknown territory. The aerosol (aqueous droplet) was suggested to be a plausible origin of life (Dobson *et al*. 2000; Griffith & Vaida, 2012). Indeed, the reactions of life systems mostly occur in confined microdroplet-like environments such as cells and cellular organelles. It is well known that macromolecular crowding alters the properties of molecules in a solution when high concentrations of macromolecules such as proteins are present (Ellis, 2001; Zhou *et al*. 2008). We envision that micro-droplet chemistry may lead to a better understanding of the chemistry in confined environments, which may be relevant to biochemical processes in a cell.

## Figures and Tables

**Table 1:** ESI-MS signal intensities of different protonated species (precursor **C**, intermediate **D,** and product **E**) obtained from different solvents^a^

Solvent	Counts in detector				

	C	D	E	[E/C] × 10^3^	[D/C] × 10^3^
Water	1·42 × 10^7^	2·12 × 10^6^	9·77 × 10^4^	6·88	149·29
Methanol	2·15 × 10^7^	3·60 × 10^6^	1·60 × 10^5^	7·44	167·44
ACN/DMF (1:1; v/v)^b^	4·86 × 10^5^	6·00 × 10^4^	1·62 × 10^3^	3·33	123·46
1% (v/v) *m*-NBA in water^c^	5·98 × 10^6^	1·01 × 10^6^	5·00 × 10^4^	8·36	168·89

a Data are highly reproducible and averaging of 1 min acquisition data is presented here. Signal intensities depend on concentration as well as ionization efficiency of the analyte.

b Mixture of ACN and DMF was used as DMF (low volatile) alone is not recommended for ESI.

c *m*-Nitrobenzyl alcohol (*m*-NBA).

**Table 2:** Physical properties of different electrospray solvents^a^

Solvent	Vapor pressure at 20 °C (torr)	Boiling point (°C)	Surface tension (mN m^−1^)
Water	17·5	100	72
Methanol	87·9	65	22
ACN	72·8	81	29
DMF	2·7	152	37
*m*-NBA^b^	<1·9	175–180	50

a Data taken from (Lide, 1996).

b Data taken from (Banerjee, 2013).
